# Imbalanced sleep increases mortality risk by 14–34%: a meta-analysis

**DOI:** 10.1007/s11357-025-01592-y

**Published:** 2025-03-12

**Authors:** Zoltan Ungvari, Mónika Fekete, Péter Varga, János Tibor Fekete, Andrea Lehoczki, Annamaria Buda, Ágnes Szappanos, György Purebl, Anna Ungvari, Balázs Győrffy

**Affiliations:** 1 Vascular Cognitive Impairment, Neurodegeneration and Healthy Brain Aging Program, Department of Neurosurgery, University of Oklahoma Health Sciences Center, Oklahoma City, OK USA; 2 Stephenson Cancer Center, University of Oklahoma, Oklahoma City, OK USA; 3 Oklahoma Center for Geroscience and Healthy Brain Aging, University of Oklahoma Health Sciences Center, Oklahoma City, OK USA; 4 Department of Health Promotion Sciences, College of Public Health, University of Oklahoma Health Sciences Center, Oklahoma City, OK USA; 5 International Training Program in Geroscience, Doctoral College, Health Sciences Program/Institute of Preventive Medicine and Public Health, Semmelweis University, Budapest, Hungary; 6 Institute of Preventive Medicine and Public Health, Semmelweis University, Budapest, Hungary; 7 Doctoral College, Health Sciences Program, Semmelweis University, Budapest, Hungary; 8 Department of Bioinformatics, Semmelweis University, 1094 Budapest, Hungary; 9 Cancer Biomarker Research Group, Institute of Molecular Life Sciences, HUN-REN Research Centre for Natural Sciences, 1117 Budapest, Hungary; 10 Department of Vascular and Endovascular Surgery, Heart and Vascular Center, Semmelweis University, Budapest, Hungary; 11 Department of Rheumatology and Clinical Immunology, Semmelweis University, Budapest, Hungary; 12 Institute of Behavioural Sciences, Semmelweis University, Budapest, Hungary; 13 Department of Biophysics, Medical School, University of Pecs, 7624 Pecs, Hungary

**Keywords:** Longevity, Meta-analysis, Survival, Mortality, Sex difference, Clinical trials, Semmelweis Study

## Abstract

Sleep duration is a crucial factor influencing health outcomes, yet its relationship with mortality remains debated. In this meta-analysis, we aimed to investigate the association between short and long sleep duration and all-cause mortality in adults, including sex-specific differences. A systematic search was performed in multiple databases, including PubMed, Cochrane Central, and Web of Science, up to October 2024. Retrospective and prospective cohort studies involving adults with at least 1 year of follow-up and data on sleep duration and all-cause mortality were included. Hazard ratios were pooled using a random-effects model, with subgroup analyses performed based on sex and sleep duration categories. A total of 79 cohort studies were included, with data stratified by sex and categorized into short and long sleep durations. Short sleep duration (< 7 h per night) was associated with a 14% increase in mortality risk compared to the reference of 7–8 h, with a pooled hazard ratio of 1.14 (95% CI 1.10 to 1.18). Conversely, long sleep duration (≥ 9 h per night) was associated with a 34% higher risk of mortality, with a hazard ratio of 1.34 (95% CI 1.26 to 1.42). Sex-specific analyses indicated that both short and long sleep durations significantly elevated mortality risk in men and women, although the effect was more pronounced for long sleep duration in women. Both short and long sleep durations are associated with increased all-cause mortality, though the degree of risk varies by sex. These findings underscore the importance of considering optimal sleep duration in public health strategies aimed at enhancing longevity and highlight the need for sex-specific approaches in sleep health research.

## Introduction

Sleep deprivation has emerged as a pervasive health challenge, affecting millions globally and leading to a wide range of serious health implications. Epidemiologically, inadequate sleep—defined as fewer than 7 h per night—is increasingly recognized as a widespread concern linked to chronic illness, accelerated aging, and increased mortality. Recent estimates indicate that up to one-third of adults regularly experience insufficient sleep, driven by lifestyle factors deeply rooted in modern society [1–3]. The demands of shift work [3, 4], intensified workplace pressures, heightened stress levels, and the pervasive influence of digital devices have all contributed to this epidemic. The allure of screens, particularly through social media, streaming platforms, and video games, often delays sleep onset and disrupts overall sleep quality. Excessive screen exposure, especially before bedtime, interferes with natural circadian rhythms through blue light exposure, further entrenching the cycle of insufficient sleep [5].

The health effects of inadequate sleep are profound and wide-ranging [6–8]. Chronic sleep deprivation has been linked to physiological impairments including weakened immune response [9], cognitive decline, cardiovascular disease [10], and metabolic dysregulation [11]. These impairments not only increase the risk of age-related diseases but also accelerate biological aging, contributing to unhealthy aging and the pathogenesis of a wide range of age-related conditions such as dementia [12, 13], diabetes [11], and cancer [14–16]. Mechanistically, chronic sleep deprivation triggers increased inflammation [17], oxidative stress [18], and hormonal imbalances [16], which collectively disrupt cellular homeostasis and tissue repair. As a result, the cumulative impact of inadequate sleep contributes to premature biological aging [19–21], heightening the likelihood of early mortality.

Despite the growing recognition of inadequate sleep as a major health risk, gaps remain in understanding the full extent of its role in mortality and disease progression. While previous studies highlight the link between inadequate sleep, individual health conditions and mortality [22, 23], comprehensive evidence connecting inadequate sleep to overall mortality risk from the perspective of unhealthy aging remains limited. Addressing this gap is crucial, as understanding the relationship among age-related sleep pathology, aging, and mortality could enhance public health approaches to mitigate aging-related health risks.

Following the pioneering work of Cappuccio et al., 15 years after their landmark publication [22], this meta-analysis aims to provide an updated evaluation of the effects of inadequate sleep on mortality risk, with a specific focus on its association with unhealthy aging. By examining a range of studies on sleep duration and mortality, we seek to provide a comprehensive, evidence-based perspective on the connection between insufficient sleep and health decline. In light of these findings, we aim to emphasize the often underrecognized role of inadequate sleep as a critical public health issue, underscoring the need for targeted interventions that promote healthier sleep habits as a preventive strategy to mitigate aging-related health risks and improve overall population health.

## Methods

### Study design and eligibility criteria

We included both retrospective and prospective cohort studies involving adult participants, with the primary outcome being all-cause mortality as defined by each individual study. A minimum follow-up period of 1 year was required for all studies. No restrictions were placed on language or other aspects of the studies. Eligible studies focused on adults aged 18 years or older, and utilized either retrospective or prospective cohort designs. The studies had to report on the relationship between sleep duration and all-cause mortality, and meet the 1-year follow-up criterion. Studies that included pregnant women or participants under 18 years of age, as well as case–control or cross-sectional studies, were excluded. Additionally, we excluded research that lacked adequate data on sleep duration or mortality outcomes, or that did not meet peer-review standards or display sufficient methodological rigor. Previous meta-analyses investigating these issues were also included [22, 24–28].

### Literature search strategy

A comprehensive literature search was conducted using medical terminology related to sleep duration and mortality, incorporating both Medical Subject Headings (MeSH) and free-text keywords. The search strategy included phrases such as “sleep duration and mortality,” “sleep duration and all-cause mortality,” “sleep duration and health outcomes,” “short sleep duration and mortality,” “long sleep duration and mortality,” and “sleep duration and longevity.” Databases searched included PubMed, Web of Science, Cochrane Central Register of Controlled Trials (CENTRAL), and Google Scholar, with the search spanning from the inception of these databases up to October 2024. In order to ensure a thorough review, we also examined references from relevant articles and considered additional sources such as conference abstracts, thesis databases, and clinical trial repositories like clinicaltrials.gov.

**Fig. 1:**
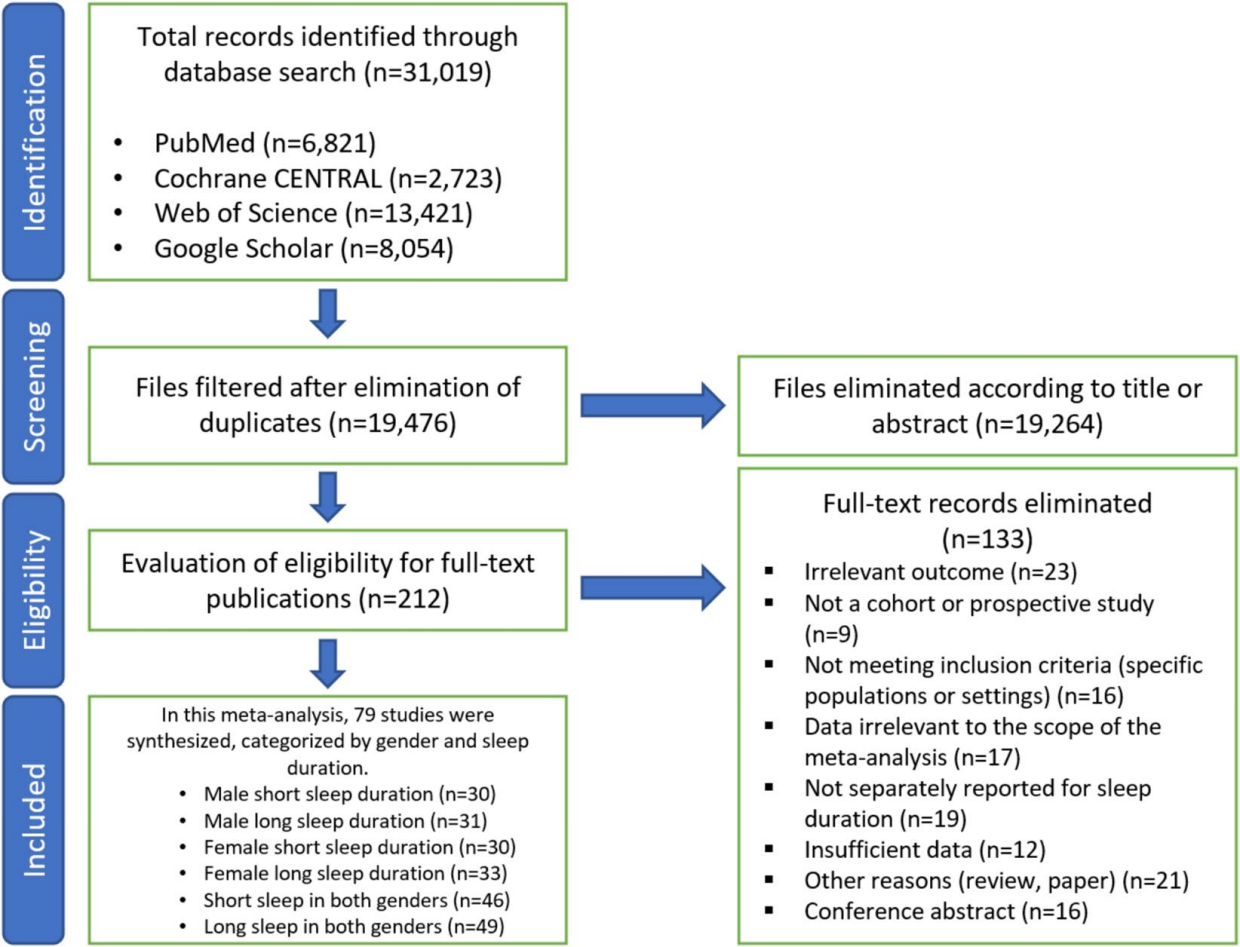
In our meta-analysis, we initially identified 31,019 records, narrowing them down to 212 after eliminating duplicates and irrelevant titles or abstracts. After further evaluation, 133 studies were excluded due to reasons such as irrelevant outcomes or insufficient data, leaving 79 studies that met our inclusion criteria. These studies included analyses of short and long sleep duration in both men and women, ensuring a comprehensive assessment of the relationship between sleep duration and mortality risk

We initially screened studies by reviewing their titles and abstracts. Full-text reviews were subsequently performed for studies that met the initial screening criteria. The predetermined inclusion and exclusion criteria were applied during this process, and data extraction was conducted based on the relevant study details. Any disagreements between reviewers during the selection or extraction process were resolved by consensus. The steps of the literature search are summarized in Fig. 1.

### Statistical methodology

Computational analyses were executed utilizing the MetaAnalysisOnline.com web-based tool [29]. To compute consolidated risk estimates (hazard ratios (HRs)) and corresponding 95% confidence intervals (CIs), we implemented a random-effects framework, which accounts for inherent variations between investigations and strengthens the broader applicability of our findings. We constructed graphical forest plots to illustrate both individual study findings and the aggregate effect, facilitating visual interpretation of the data distribution and enabling identification of inter-study disparities.

The presence of between-study variation was evaluated through two complementary approaches: Cochran’s *Q* test (chi-square analysis) and the *I*^2^ metric. While the *Q* test determined whether effect size variability exceeded random chance expectations, the *I*^2^ parameter quantified the percentage of overall variation attributable to genuine heterogeneity rather than sampling fluctuations.

### Publication bias evaluation

We investigated potential reporting bias through visual inspection of funnel diagrams, which plot effect magnitude against precision measures to identify asymmetric patterns. Additionally, we employed Egger’s regression analysis to quantitatively assess the relationship between study results and their associated precision metrics.

### Trial sequential analysis

We performed trial sequential analysis (TSA) using the *metacoumbounds* package within Stata version 14.1 to assess the cumulative evidence strength and determine the conclusiveness of our findings. The required a priori information size (APIS), representing the threshold sample size necessary for statistical significance, was calculated assuming a 15% relative risk reduction, type I error rate (α) of 5%, and statistical power of 80%.

### Subgroup analysis

Subgroup analyses were performed based on sex and specific sleep duration intervals. Sleep durations were categorized as ≤ 6–7 h and ≥ 8–9 h per night, with 7–8 h serving as the reference category for comparison in the entire study.

## Results

### Short sleep duration and mortality risk

**Fig. 2:**
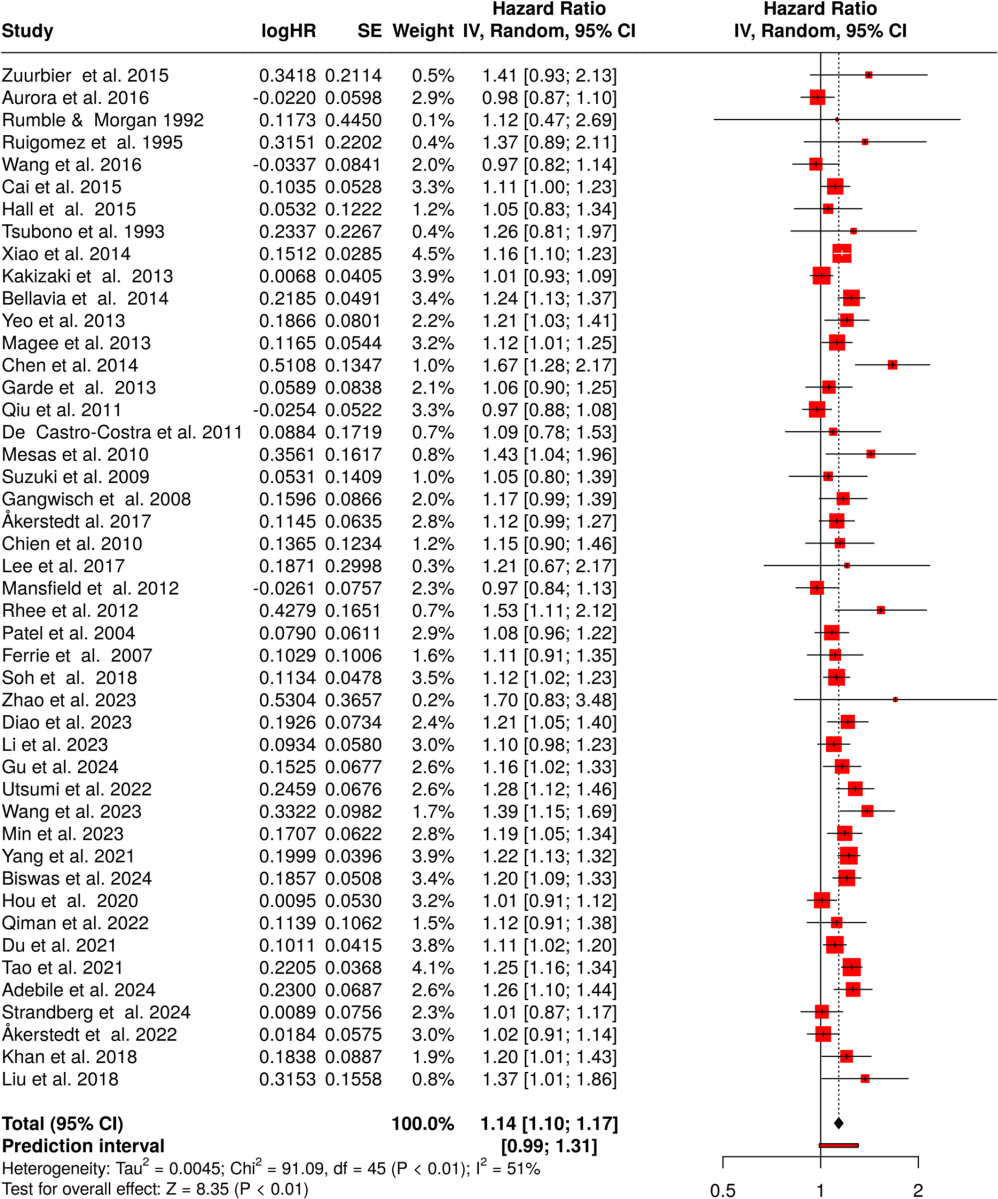
Short sleep duration and mortality risk. The forest plot depicts the hazard ratios for the association between short sleep duration and mortality, summarizing results from 46 studies. The pooled HR, calculated using a random-effects model via the inverse variance method, shows a statistically significant 14% increased risk of mortality for individuals with short sleep duration. Notably, substantial heterogeneity was observed among studies, with an *I*^2^value of 53%, indicating moderate variability in the results. Abbreviations: HR, hazard ratio; CI, confidence interval; SE, standard error; IV, inverse variance [30–75]

In the analysis of the relationship between short sleep duration and mortality, a total of 46 studies were included. The random effects model, applied with the inverse variance method to assess the pooled hazard rates, revealed a statistically significant association between short sleep duration and increased risk of mortality. The overall hazard ratio was calculated to be 1.14, with a 95% confidence interval of 1.10 to 1.17. This indicates that individuals with shorter sleep durations had a 14% higher risk of mortality compared to those with sufficient sleep. The overall effect was confirmed to be significant, with a *p*-value less than 0.05 (Fig. 2) [30–75].

However, the analysis also detected considerable heterogeneity among the studies, with a *p*-value below 0.01. This suggests variability in the magnitude and potentially the direction of the effect across different cohorts. The *I*^2^ statistic was 51%, meaning that just over half of the variability between studies was attributable to differences in study populations, methodologies, or other factors, rather than random variation.

**Fig. 3:**
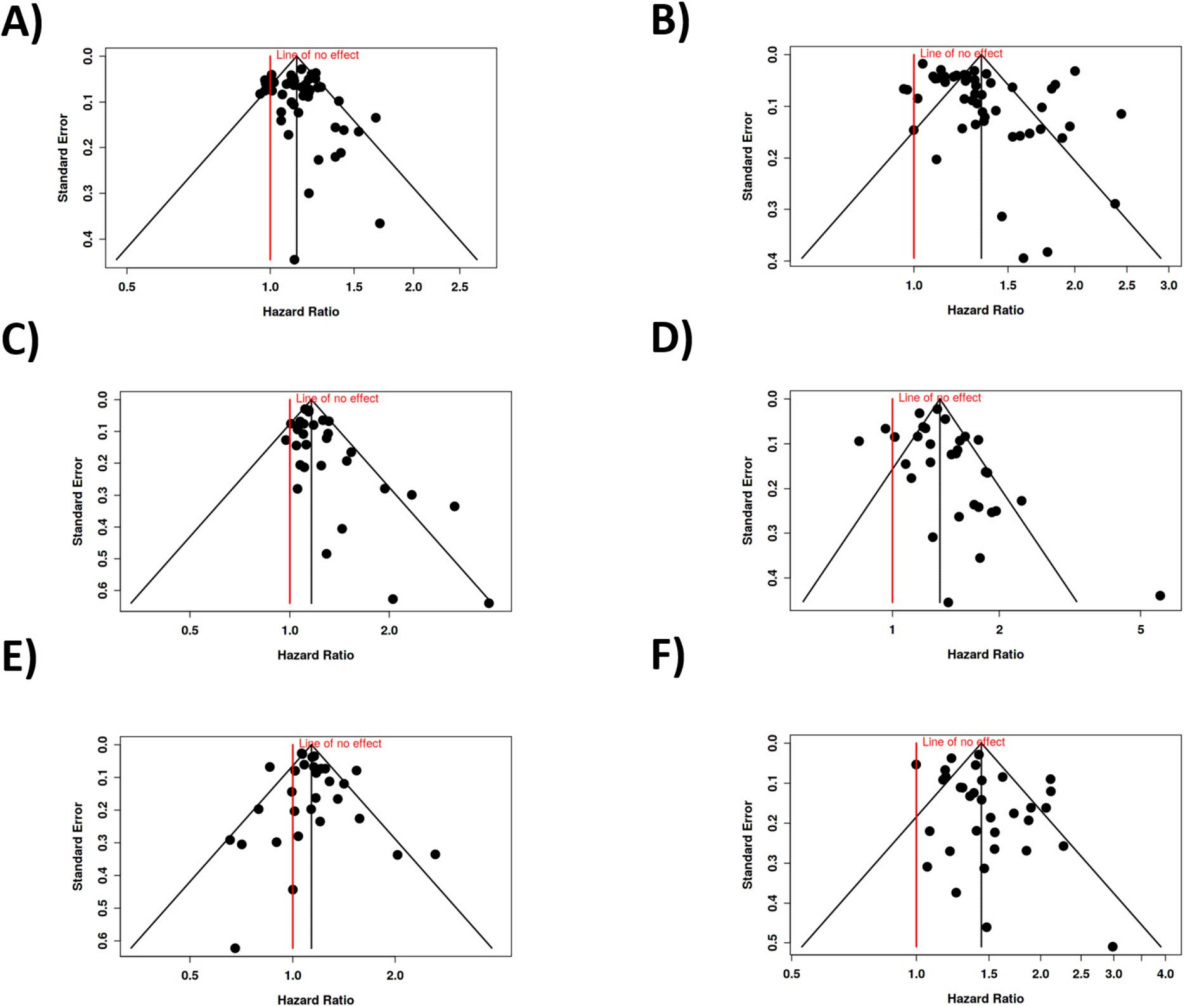
Funnel plots to assess potential publication bias in meta-analyses of sleep duration and mortality risk across different subgroups. The plots depict the distribution of hazard ratios against standard errors for studies included in the meta-analyses. **A**,** B** The results for both sexes: short sleep duration (**A**) and long sleep duration (**B**). Egger’s test detected significant asymmetry in **B**, indicating possible publication bias, while **A** showed no significant asymmetry. Panels **C** and **D** focus on men: short sleep duration (**C**) and long sleep duration (**D**). Both analyses showed significant asymmetry (Egger’s test), suggesting possible publication bias in these subgroups. Panels **E** and** F** depict women: short sleep duration (**E**) and long sleep duration (**F**). Neither plot showed significant asymmetry based on Egger’s test, indicating no evident publication bias in these analyses. The red line represents the ‟line of no effect” (HR = 1), while the dotted lines outline the expected distribution in the absence of bias

In terms of publication bias, the funnel plot did not suggest any significant skewing of the data. This finding was further supported by Egger’s test, which did not reveal any funnel plot asymmetry (intercept, 0.52; 95% CI, − 0.36–1.4; *t*, 1.154; *p*-value, 0.255; see Fig. 3A). These results suggest that the likelihood of smaller or negative studies being underreported in the literature is minimal.

### Long sleep duration and mortality

In our second analysis, comprising 49 studies, we examined the association between long sleep duration and mortality. A random effects model was employed to synthesize HRs from these studies.

**Fig. 4:**
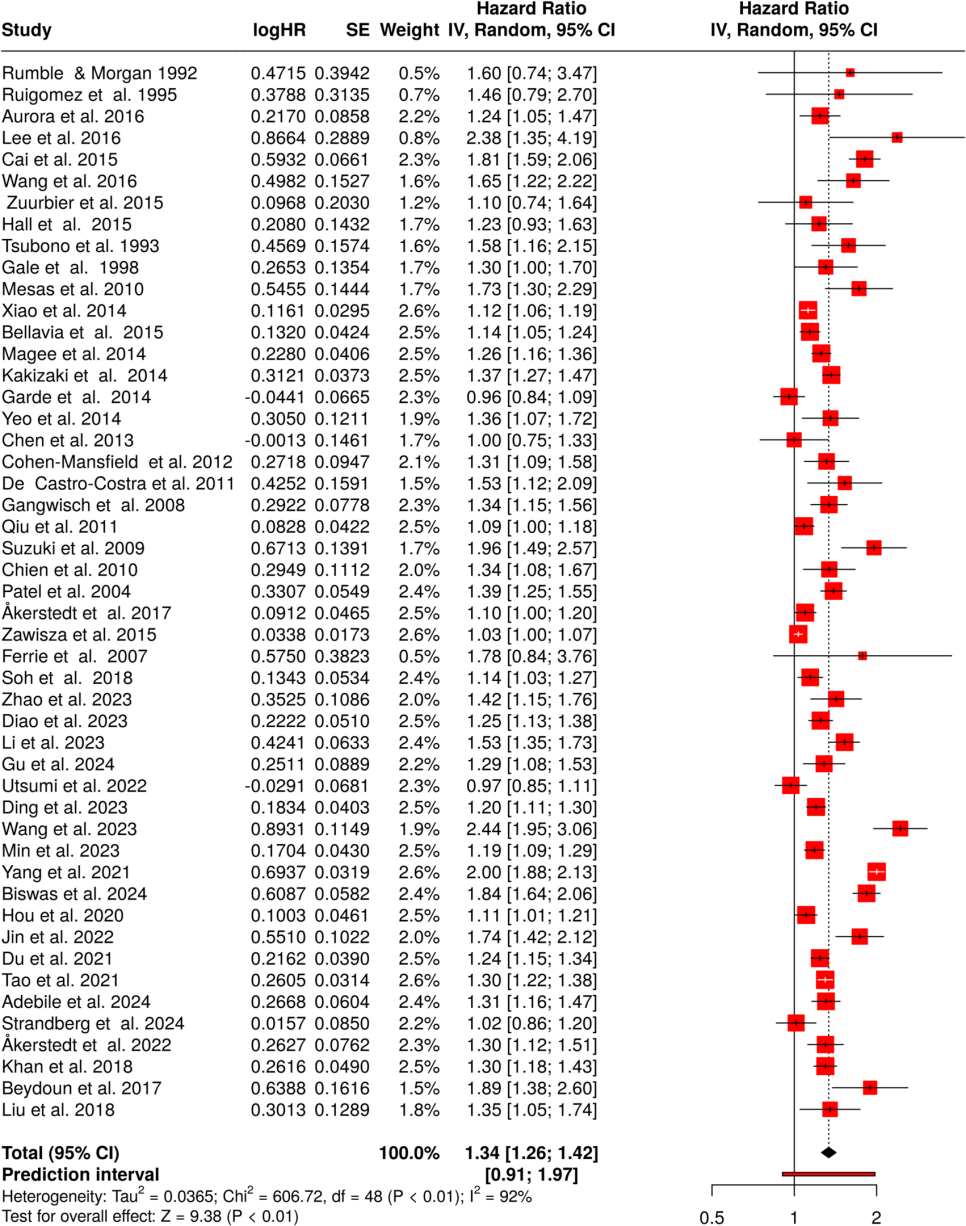
Long sleep duration and mortality. The forest plot presents the hazard ratios for the association between long sleep duration and mortality, derived from 49 studies. The random-effects model applied through the inverse variance method revealed a statistically significant 34% increased risk of mortality for individuals with long sleep duration. Notably, significant heterogeneity was detected (*I*^2^= 92%), indicating substantial variability in the study results. Abbreviations: HR, hazard ratio; CI, confidence interval; SE, standard error; IV, inverse variance [30–40, 42–79]

The pooled analysis revealed a statistically significant positive association between long sleep duration and mortality. The summary HR was 1.34 (95% confidence interval, 1.26–1.42), indicating that individuals with long sleep duration had a 34% higher risk of mortality compared to those with shorter sleep (Fig. 4) [30–40, 42–79]. This finding was supported by a significant overall effect (*p* < 0.05).

However, substantial heterogeneity was observed among the studies (*p* < 0.01). This suggests that the magnitude and/or direction of the effect varied across different studies. The *I*^2^ value of 92% indicates that the majority of the variability among studies was due to heterogeneity rather than random chance. Furthermore, the funnel plot analysis and Egger’s test suggested the potential presence of a publication bias. The funnel plot asymmetry and significant Egger’s test result (intercept, 2.08; 95% CI, 0.39–3.77; *t*, 2.407; *p*-value, 0.02, visualized in Fig. 3B) indicate that studies with smaller effect sizes might be underrepresented in the literature.

### Sleep duration and mortality in males

**Fig. 5:**
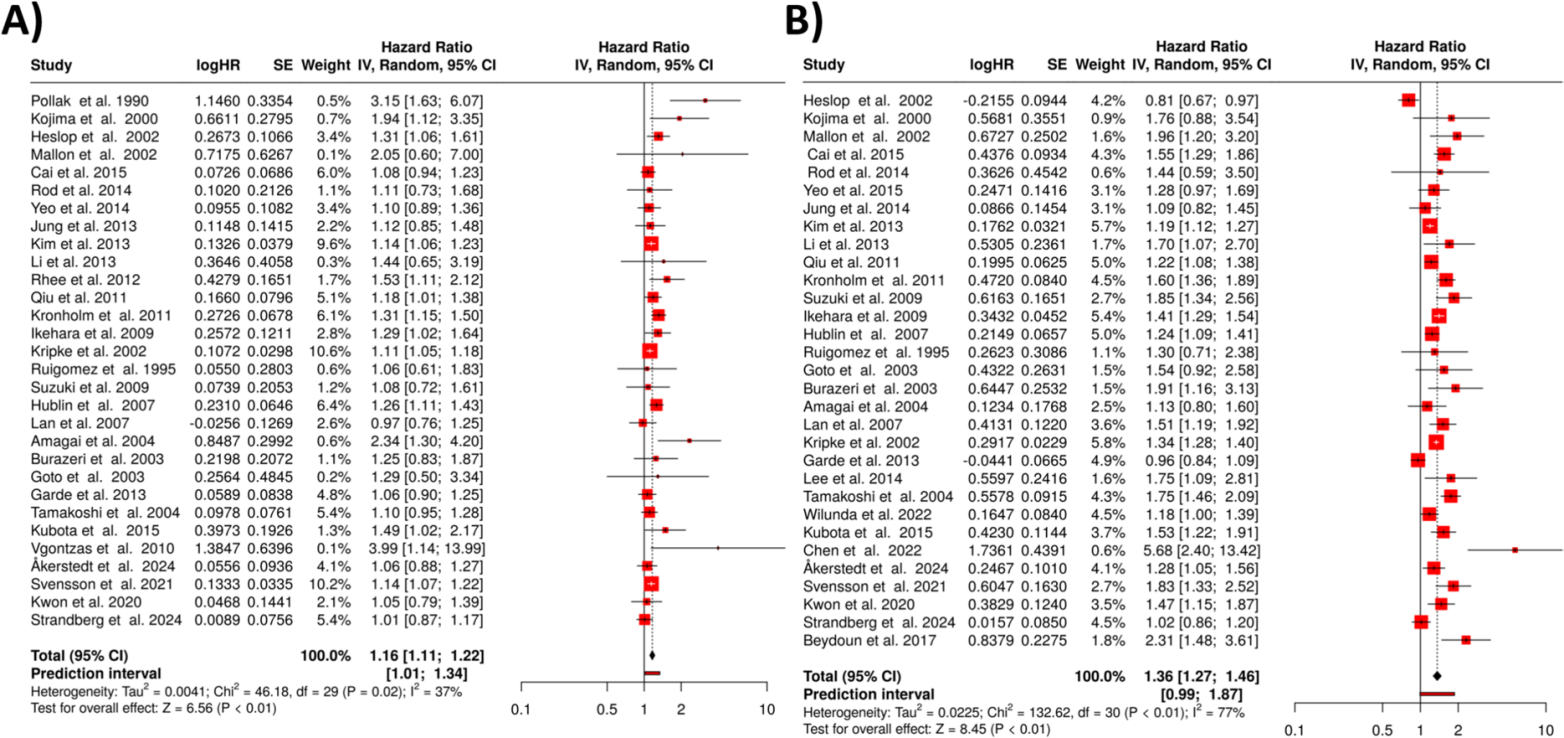
Sleep duration and mortality in males. This figure presents the results of two meta-analyses examining the association between sleep duration and mortality in males. **A** The analysis for short sleep duration (< 7 h), including 30 studies, and shows a statistically significant 16% increase in mortality risk. Heterogeneity among studies was modest (*I*^2^ = 37%). **B** The analysis for long sleep duration (≥ 8 h), based on 31 studies, revealing a statistically significant 36% increase in mortality risk. Heterogeneity in this analysis was substantial (*I*^2^= 77%). Abbreviations: HR, hazard ratio; CI, confidence interval; SE, standard error; IV, inverse variance [33, 37, 41, 44, 49, 50, 54, 72, 79–104]

In the third setting, only those studies were included, where outcome in males were reported. In the meta-analysis of *short sleep duration* (less than 7 h), 30 studies were included in the final analysis (Fig. 5A). Using a random effects model with the inverse variance method to compare the hazard rates, the results revealed a statistically significant association between short sleep and increased mortality risk. The overall hazard ratio was calculated to be 1.16, with a 95% confidence interval of 1.11 to 1.22. This indicates that individuals with shorter sleep durations face a 15% increased risk of mortality compared to those with adequate sleep. The test for overall effect was significant at *p* < 0.05.

Despite this association, heterogeneity among the studies was observed, with a *p*-value of 0.02 and an *I*^2^ statistic of 37%. This suggests that while the overall effect is significant, there is variability in the magnitude and direction of the effect across different studies, although this heterogeneity is relatively modest. The funnel plot indicated the presence of potential publication bias, which was further supported by Egger’s test (intercept, 0.92; 95% CI, 0.28–1.56; *t*, 2.818; *p*-value, 0.009; depicted in Fig. 3C), suggesting that smaller studies with negative or less dramatic results may be underreported.

For *long sleep duration* (8–9 h or more), 31 studies were analyzed (Fig. 5B). Similarly, the random effects model with the inverse variance method demonstrated a statistically significant association between long sleep duration and increased mortality risk. The overall hazard ratio was 1.36, with a 95% confidence interval of 1.27 to 1.46, indicating a 36% higher mortality risk among individuals with extended sleep durations. The test for overall effect was significant at *p* < 0.05.

However, in contrast to short sleep duration, the heterogeneity in the long sleep analysis was more pronounced, with a *p*-value of less than 0.01 and an *I*^2^ value of 77%, indicating substantial variability across studies. This suggests that the effects of long sleep duration on mortality risk are more inconsistent in magnitude and possibly direction across different studies. The funnel plot suggested a potential publication bias, and this was supported by Egger’s test (intercept, 0.91; 95% CI, − 0.19–2.02; *t*, 1.615; *p*-value, 0.117; see Fig. 3D).

### Sleep duration and mortality in females

**Fig. 6:**
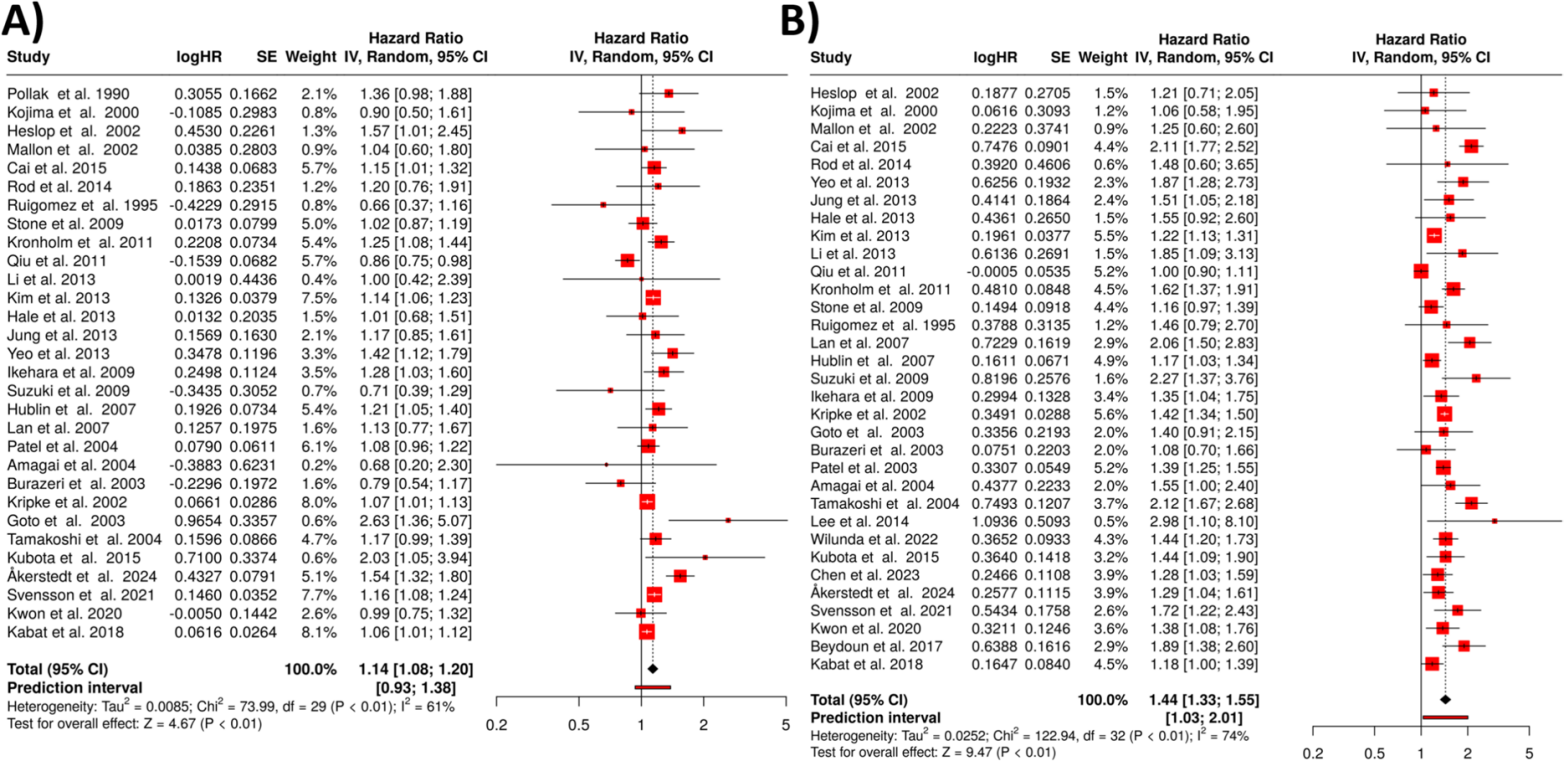
Sleep duration and mortality in females according to the results of two meta-analyses. **A** The analysis of short sleep duration (< 7 h), including 30 studies, which found a 14% increased mortality risk. Heterogeneity was moderate (*I*^2^ = 61%), suggesting that meaningful differences exist among the studies. **B** The analysis of long sleep duration (≥ 8 h), based on 33 studies, revealing a 44% increased mortality risk. Heterogeneity in this analysis was substantial (*I*^2^= 74%), indicating greater variability across the studies. Abbreviations: HR, hazard ratio; CI, confidence interval; SE, standard error; IV, inverse variance [33, 34, 37, 49, 50, 54, 79–88, 90–108]

We identified and evaluated 30 studies investigating the relationship between *short sleep duration* and mortality risk in women (Fig. 6A). The meta-analysis demonstrated that women who reported short sleep duration had a 13% higher mortality risk compared to those with normal sleep duration. The summarized hazard rate was 1.14 (95% confidence interval, 1.08 to 1.20), with the overall effect showing statistical significance (*p* < 0.05).

The analysis revealed moderate heterogeneity among the included studies (*p* < 0.01). The *I*^2^ statistic of 61% indicates that more than half of the observed variation in effect sizes across studies was attributable to true heterogeneity rather than random chance, suggesting meaningful differences in the magnitude and/or direction of effects across the analyzed studies. The definition of short and long sleep duration varied across the included studies, with thresholds for short sleep ranging from ≤ 6 to ≤ 7 h and for long sleep from ≥ 8 to ≥ 9 h. This variability likely contributed to the observed heterogeneity in the pooled hazard ratios. Standardizing these definitions across future studies would improve comparability and enhance the reliability of meta-analytic estimates.

To evaluate potential publication bias, we conducted both visual and statistical assessments. The funnel plot exhibited symmetrical distribution of effect sizes, suggesting no evident publication bias. This observation was further corroborated by Egger’s regression test, which showed no significant funnel plot asymmetry (intercept, 0.36; 95% CI, − 0.51–1.23; *t*, 0.814; *p*-value, 0.423; visualized in Fig. 3E).

We identified and evaluated 33 studies examining the association between *long sleep* duration (8–9 + h) and mortality risk in women (Fig. 6B). The meta-analysis, conducted using a random effects model with inverse variance weighting, revealed that women who reported long sleep duration had a 41% higher mortality risk compared to those with normal sleep duration. Specifically, the summarized hazard rate was 1.44 (95% confidence interval, 1.33 to 1.55), indicating a statistically significant association (*p* < 0.05).

Our analysis detected substantial heterogeneity among the included studies (*p* < 0.01). The calculated *I*2 statistic of 74% suggests that nearly three-quarters of the observed variation in effect sizes across studies was attributable to true heterogeneity rather than random chance, indicating meaningful differences in the magnitude and/or direction of effects across studies.

To assess potential publication bias, we conducted both visual and statistical evaluations. The funnel plot appeared symmetrical, suggesting no obvious publication bias. This observation was supported by Egger’s regression test, which did not indicate significant funnel plot asymmetry (intercept, 0.94; 95% CI, − 0.09–1.97; *t*, 1.795; *p*-value, 0.082; provided in Fig. 3F).

### Results of trial sequential analysis

**Fig. 7:**
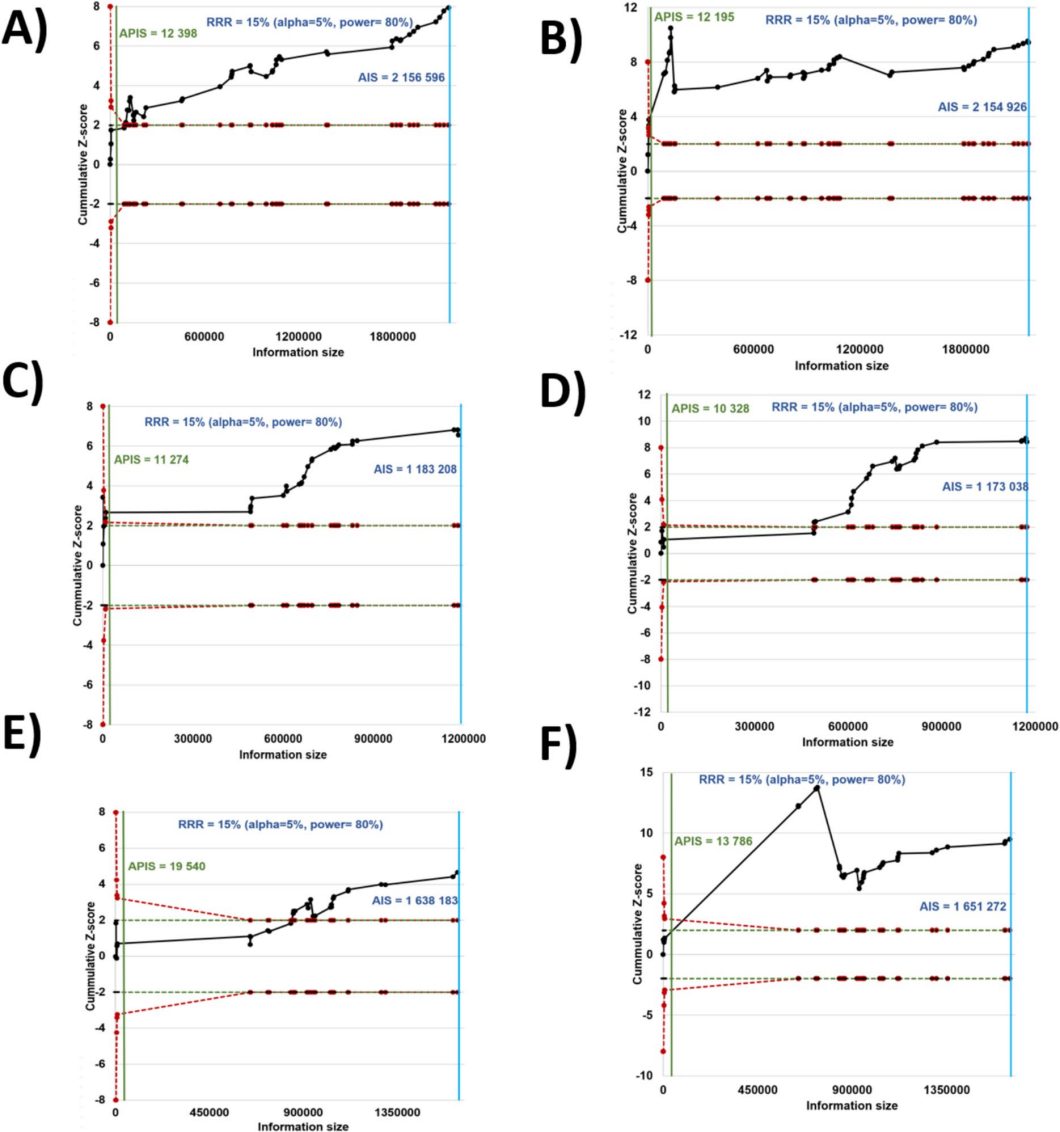
The trial sequential analysis (TSA) plots illustrate cumulative *Z*-scores for short and long sleep durations in both sexes, as well as separated by male and female subgroups. Panels **A** and** B** represent both sexes, **C** and** D** show results for males, and **E** and** F** for females. A required a priori information size (APIS) is indicated to determine if enough data have been collected to draw conclusions. In all plots, the actual information size (AIS), represented by the blue lines surpasses the APIS

The trial sequential analysis (TSA) results for both short and long sleep duration in relation to mortality risk, presented in Fig. 7, indicate that the actual information size (AIS) surpasses the adjusted required a priori information size (APIS) across all panels (A–F). This demonstrates that the sample size is more than sufficient for reliable conclusions in all subgroups, regardless of sex or sleep duration. In other words, the TSA plots show that the accumulated evidence is statistically robust, suggesting that further trials are not necessary to determine the relationship between sleep duration (both short and long) and mortality risk.

## Discussion

This meta-analysis demonstrates that inadequate sleep, specifically sleeping less than 7 h per night, is associated with a 14% increased risk of all-cause mortality. These findings underscore inadequate sleep as an important risk factor for mortality, particularly among adults in modern societies where sleep deprivation is increasingly prevalent.

Our findings extend the results of numerous prior studies that have established a link between short sleep duration and increased mortality risk [22, 24–28]. Interestingly, short sleep duration has been linked to a slightly higher mortality risk in men, possibly due to a greater incidence of common public health concerns as sleep apnea, cardiovascular disease, and lifestyle factors like smoking and alcohol use, which are more common in men and may compound the adverse effects of insufficient sleep. This discrepancy might also reflect sex differences in sleep architecture, hormonal regulation, and other health conditions that contribute to altered sleep duration. These findings highlight the importance of considering sex-specific factors when evaluating sleep-related mortality risks, as biological and lifestyle differences can shape sleep’s impact on health and longevity. While the analyzed studies did not specify causes of mortality, previous research has consistently shown that insufficient sleep correlates with various health risks, including accelerated aging processes [17, 19–21], cardiovascular disease [10], metabolic syndrome [11], cancer [14–16], cognitive decline and dementia [12, 13, 109], and compromised immune function [9]. Any of these factors could potentially contribute to the increased mortality risk associated with inadequate sleep.

The physiological basis for the association between inadequate sleep and increased mortality risk may involve multiple biological pathways. A growing body of evidence suggests that inadequate sleep accelerates various biological processes associated with aging, contributing to physiological decline and elevated mortality risk. Sleep is integral to cellular repair and maintenance, and chronic sleep deprivation is linked to several hallmarks of aging [110], including increased oxidative stress [18], heightened inflammation [17], cellular senescence [19, 111, 112], adverse changes in metabolism [113, 114], and epigenetic changes [20, 21, 115]. Inadequate sleep contributes to oxidative stress by impairing mitochondrial function [116–122], leading to an overproduction of reactive oxygen species that can damage cellular DNA, proteins, and lipids. Furthermore, sleep loss results in a chronic low-grade inflammation [17] that is implicated in age-related diseases such as cardiovascular disease and dementia. At the cellular level, insufficient sleep impairs telomere function [19, 111, 112] promoting the accumulation of senescent cells, which secrete inflammatory factors and further exacerbate tissue damage and aging and the pathogenesis of age-related diseases [123–126]. Additionally, sleep influences epigenetic regulation by affecting DNA methylation [20, 21, 115] and histone modification patterns, processes that are crucial for maintaining genomic stability and proper gene expression. Collectively, these disruptions highlight how chronic sleep insufficiency may accelerate biological aging and increase vulnerability to various age-related diseases.

Inadequate sleep has been shown to increase blood pressure [127–130], influence heart rate variability [131], and elevate inflammatory markers [132, 133], all of which are linked to cardiovascular disease [134]. The resultant strain on the cardiovascular system from chronic sleep deprivation is believed to contribute significantly to heightened mortality risk, as cardiovascular events remain a leading cause of death worldwide.

The glymphatic system serves as the brain’s waste clearance pathway, efficiently removing neurotoxic substances like amyloid-beta (Aβ) proteins, which are central to Alzheimer’s disease pathology [135]. Deep, restorative sleep is crucial for glymphatic function, increasing clearence of Aβ [136–138]. Chronic sleep deprivation disrupts this clearance process, leading to Aβ buildup and promoting the pathogenesis of Alzheimer’s disease [136–138].

Chronic sleep deprivation has also been associated with adverse metabolic outcomes, including insulin resistance, weight gain, and dyslipidemia [11, 113]. Short sleep duration disrupts glucose metabolism and increases appetite-regulating hormones, leading to elevated risks of obesity, diabetes [139], and other metabolic disorders that contribute to mortality risk [11, 140, 141]. Poor sleep is also associated to unfavourable changes of cholesterol metabolism [114].

Inadequate sleep can also have a direct impact on mental health and cognitive function, contributing to accelerated cognitive decline, mood disorders, and heightened stress response [3, 142–144]. Sleep deprivation impairs the brain’s ability to consolidate memory, regulate emotions, and manage stress, which indirectly affects mortality risk through increased risk of accidents, mental health disorders, and impaired decision-making [118, 145].

Additionally, inadequate sleep may contribute to an increased risk of cancer [14–16], though the relationship is complex and varies depending on cancer type, sleep patterns, and other lifestyle factors. The mechanisms by which inadequate sleep is thought to promote cancer risk include immune system suppression, hormonal disruptions, heightened state of inflammation [146], metabolic dysregulation, and circadian rhythm disruption. First, inadequate sleep weakens immune function, which is crucial for identifying and destroying cancer cells. Chronic sleep deprivation can reduce the activity of natural killer cells, a type of immune cell that helps detect and eliminate tumor cells early on. Second, sleep deprivation alters the balance of hormones like melatonin, cortisol, and insulin. Melatonin, a hormone produced during sleep, has antioxidant properties and helps regulate cell growth [16]. Lower levels of melatonin, as seen in sleep-deprived individuals, may leave cells more vulnerable to mutations and tumor growth. Third, inadequate sleep is linked to increased levels of inflammation, a condition associated with the development and progression of cancer. Chronic inflammation can damage cellular DNA, increasing the likelihood of mutations that lead to cancer. Fourth, poor sleep impacts insulin sensitivity and promotes weight gain and obesity, both of which are associated with higher risks of cancers [147], including breast, colorectal, and prostate cancer. Fifth, the body’s natural circadian rhythm is closely tied to cell cycles and DNA repair processes [148, 149]. Shift workers, who frequently experience circadian rhythm disruptions, show an increased risk of cancers, particularly breast, thyroid, and prostate cancer [16, 150–152]. This effect is believed to be due in part to the continuous desynchronization between the body’s internal clock and environmental light–dark cycles, leading to prolonged exposure to growth-promoting factors.

While this study focused on sleep duration, sleep quality is another critical factor influencing mortality risk. Poor sleep quality, characterized by frequent awakenings, reduced deep sleep, and sleep fragmentation, has been associated with cardiovascular disease, impaired glymphatic clearance, and neurodegeneration. Future research should investigate how sleep quality interacts with duration to influence long-term health outcomes.

Inadequate sleep is increasingly recognized as a significant, yet often underdiagnosed, public health issue with profound implications for health and longevity. This study emphasizes the need for public health policies that prioritize sleep health alongside other well-established lifestyle factors, such as diet [153], exercise, and smoking cessation. Population-level interventions should prioritize sleep health education and interventions tailored to vulnerable groups, such as shift workers and individuals with pre-existing health conditions, to reduce mortality risks associated with inadequate or excessive sleep. Addressing inadequate sleep as a public health priority may involve educational campaigns, workplace policies that limit shift work or provide flexibility for adequate rest, and greater awareness of the role of digital devices in sleep disruption. Moreover, integrating sleep health into routine medical assessments could aid in identifying and addressing sleep deficiencies before they contribute to severe health outcomes.

One of the important goals of our meta-analysis was to inform the design and focus of the Semmelweis Study, an ongoing research initiative led by Semmelweis University in Budapest, Hungary [154]. This study is dedicated to comprehensively exploring the factors that contribute to unhealthy aging, with a particular emphasis on the role of sleep. Recognizing sleep as a critical yet often underappreciated determinant of health, the Semmelweis Study investigates how inadequate or disrupted sleep patterns may accelerate physiological and cognitive declines associated with aging. The Semmelweis Study is uniquely positioned to examine sleep’s impact on a large, diverse workforce that includes a significant proportion of shift workers. Shift work or irregular work pattern, prevalent in healthcare, is associated with circadian rhythm disruption and chronic sleep deprivation—both of which have been linked to an increased risk of cancer [150–152], cardiovascular disease [155], metabolic disorders [156, 157], and cognitive decline [158–160]. By focusing on this population, the study aims to uncover how the demanding schedules and irregular sleep patterns of shift workers may further elevate risks for unhealthy aging and development of age-related diseases. Drawing on our meta-analysis findings, the Semmelweis Study will examine both subjective and objective measures of sleep duration, quality, and timing among shift workers and non-shift workers alike. This approach enables the study to assess the compounded health effects of inadequate sleep combined with occupational factors, such as long or irregular work hours. Additionally, the study seeks to identify modifiable behaviors that could mitigate the health impacts of shift work, and aims to apply workplace-based interventions that promote sleep hygiene, and also support circadian alignment and recovery sleep during off hours. Through this workforce-centered approach, the Semmelweis Study aims to inform public health strategies that prioritize sleep health as a key component of health promotion and healthy aging, particularly for vulnerable populations with high occupational demands. By investigating the relationship between sleep insufficiency and specific age-related health conditions, such as cardiovascular disease [161] and neurodegenerative disorders, the study seeks to highlight the need for tailored public health interventions that address the unique challenges faced by shift workers and aging individuals.

Despite the robustness of these findings, several limitations should be considered. The studies included in this meta-analysis cover a diverse range of populations, settings, and methodologies, which may introduce variability in the results. Differences in sleep definitions and measurement methods, as well as population-specific factors, could influence the generalizability of these findings. Additionally, many studies in the analysis rely on self-reported sleep duration, which is subject to recall bias and may not accurately reflect actual sleep patterns. Objective methods, such as actigraphy or polysomnography, provide more reliable measurements of sleep duration and should be prioritized in future research to validate self-reported findings. Although efforts were made to adjust for confounding variables, residual confounding may still affect the results. Factors such as socioeconomic status, pre-existing health conditions, and lifestyle habits could independently impact mortality risk and are not uniformly controlled across all included studies. Future studies should apply uniform and comprehensive confounder adjustments to better isolate the effects of sleep duration on mortality risk. Additionally, potential publication bias was assessed through funnel plot analyses, and while efforts were made to include all relevant studies, publication bias remains a limitation, as studies with null or less significant findings may be underreported. Our analysis also revealed a significant association between long sleep duration and increased mortality risk. This effect remained significant across sex and population subgroups, suggesting that extended sleep may be linked to underlying health issues that increase mortality risk, such as undiagnosed chronic illnesses or metabolic dysregulation.

To build on these findings, future studies could examine the dose–response relationship between sleep duration, sleep pathologies, and specific causes of mortality to better understand how even slight deviations from optimal sleep duration influence health outcomes. Additionally, investigating disease-specific mortality analyses would provide crucial insights, as different diseases—such as cardiovascular disease, cancer, and neurodegenerative disorders—may have unique associations with sleep duration, quality, and circadian disruption. Evaluating the effects of interventions aimed at improving sleep hygiene, such as the properly aligned exposure and limitation of blue light exposure from screens, stress management, and promoting regular sleep schedules, could also help assess their potential to reduce overall and disease-specific mortality risks.

Evaluating the effects of interventions aimed at improving sleep hygiene—such as properly timed exposure to natural light, limitation of blue light exposure from screens, effective stress management strategies, and the promotion of regular sleep schedules—could provide valuable insights into their potential to reduce overall and disease-specific mortality risks [162–164]. Chronotype has been shown to influence sleep patterns, quality, and circadian alignment, with evening-oriented individuals (“night owls”) often experiencing greater misalignment with societal schedules. This misalignment may lead to chronic sleep deprivation, particularly in those required to wake early for work or social commitments, potentially compounding health risks associated with inadequate sleep. Investigating how chronotype interacts with sleep duration could reveal whether mortality risks differ based on an individual’s biological clock.

Taken together, this meta-analysis reinforces the significance of sleep duration as a crucial factor in mortality risk, particularly highlighting inadequate sleep as a modifiable risk factor for unhealthy aging and premature death. Addressing inadequate sleep in public health initiatives may offer a vital opportunity to improve health outcomes on a broad scale. Our findings advocate for sleep health to be prioritized in preventive health strategies aimed at enhancing longevity and quality of life across populations.
